# Mitogen-Activated Protein Kinase Signaling Mediates Morphine Induced-Delayed Hyperalgesia

**DOI:** 10.3389/fnins.2019.01018

**Published:** 2019-09-20

**Authors:** Bárbara Guimaraes de Freitas, Leandro Márcio Pereira, Flávia Vianna Santa-Cecília, Natália Gabriele Hösch, Gisele Picolo, Yara Cury, Vanessa O. Zambelli

**Affiliations:** Laboratório Especial de Dor e Sinalização, Instituto Butantan, São Paulo, Brazil

**Keywords:** morphine, opioid, opioid-induced hyperalgesia, MAPK, ERK1/2, p38, JNK, CREB

## Abstract

The use of morphine, the standard opioid drug, is limited by its undesirable effects, such as tolerance, physical dependence, and hyperalgesia (increased pain sensitivity). Clinical and preclinical studies have reported development of hyperalgesia after prolonged opioid administration or after a single dose of intrathecal (i.t.) morphine in uninjured rats. However, whether a single standard systemic morphine dose is sufficient to decrease the nociceptive threshold in rats is unknown. Here, we showed that a single morphine subcutaneous injection induces analgesia followed by a long-lasting delayed hyperalgesia in uninjured and PGE2 sensitized rats. The i.t injection of extracellular signal-regulated kinase (ERK) inhibitor blocked morphine-induced analgesia, without interfering with the morphine-induced hyperalgesia. However, i.t. injection of SB20358, a p38 inhibitor and SP660125, a JNK inhibitor, decreased the morphine-induced hyperalgesia. Consistently with the behavioral data, Western Blot analysis showed that ERK is more phosphorylated 1 h after morphine, i.e., when the analgesia is detected. Moreover, phospho-p38 and phospho-JNK levels are upregulated 96 h after morphine injection, time that coincides with the hyperalgesic effect. Intrathecal (i.t.) oligodeoxynucleotide (ODN) antisense to cAMP-responsive element binding protein (CREB) attenuated morphine-induced hyperalgesia. Real-time polymerase chain reaction (RT-PCR) analysis showed that CREB downstream genes expressions were significantly up-regulated 96 h after morphine injection in spinal cord. Together, our data suggest that central ERK is involved in the analgesic and hyperalgesic effects of morphine while JNK, p38, and CREB are involved in the morphine-induced delayed hyperalgesia.

## Introduction

Opioids are effective analgesics for treating moderate to severe pain, but their use is limited due to adverse effects such as development of tolerance and paradoxical pain. Several preclinical studies have reported that administration of opioids, such as morphine, either in very high or very low doses, increases sensitivity to noxious stimuli, i.e., hyperalgesia (Angst and Clark, 2006; Lee et al., 2011). Hyperalgesia is also reported after repeated daily opioid administration (Corder et al., 2017) or after a single intrathecal injection of opioids in rats (Van Elstraete et al., 2005). Clinical studies have reported that short-term administration of an opioid can enhance hyperalgesia (Angst et al., 2003). However, whether a single systemic administration of an intermediate opioid dose (i.e., a dose that induces analgesia without compromising the overall activity, such as, locomotion or respiratory functions) induces hyperalgesia is still controversial. Therefore, studies evaluating the effect of a single injection of opioid on the nociceptive threshold are still an unmet need and relevant for clinical practice.

Mitogen-activated protein kinases (MAPK) are a family of evolutionarily conserved molecules that transduce extracellular stimuli into intracellular responses, by changing transcription as well as inducing posttranslational modifications of target proteins. There are three major members in the MAPK family: extracellular signal regulated kinase (ERK), p38, and c-Jun N-terminal kinases (JNK) (Johnson and Lapadat, 2002; Ji and Strichartz, 2004). Several lines of evidence have shown that MAPK up-regulates the pro-nociceptive systems. Activation of ERK, JNK, and p38 in the spinal cord leads to the production of inflammatory mediators that sensitize dorsal horn neurons. The increased activity in dorsal horn transforms the subsequent performance of the nociceptive pathway, by amplifying or prolonging the response to noxious inputs (hyperalgesia). Therefore, the MAPK activation in the spinal cord contributes to pain sensitization (Ji et al., 2009).

Opioids induce paradoxical pain by activating neuronal and non-neuronal cells; however the mechanisms involved in this phenomenon are not completely understood. Involvement of MAPK in tolerance and hyperalgesia after long-term morphine exposure is well documented. Functional studies showed that inhibition of MAPK alleviates pathological pain (Ji and Woolf, 2001) and morphine tolerance (Chen and Sommer, 2009), indicating that these two conditions may share similar cellular and molecular mechanisms. Moreover, chronic morphine exposure activates ERK1/2, p38, and JNK in central and peripheral nervous systems. The downstream signaling mechanisms involve the cAMP-responsive element binding protein (CREB) pathway, which regulates the expression of neuropeptides, such as CGRP and substance P. Despite the evidences showing that chronic opioid administration activates MAPK signaling pathway, the effects of an acute injection of morphine, including at doses able to induce hyperalgesia, on those kinases are unknown. Consequently, we decided to test the hypothesis that a single dose of systemic administered morphine induces a MAPK-dependent central sensitization process and subsequent hyperalgesia. We also tested whether inhibiting ERK1/2, p38, and JNK impairs morphine-induced hyperalgesia. Finally, we evaluated the effect of inhibiting CREB transcriptional factor on morphine effects.

## Materials and Methods

### Animals

Male Wistar rats (170–190 g) were used in this study. The animals were housed in a temperature-controlled (21 ± 2°C) and light-controlled (12 h/12 h light/dark cycle) environment. All of the behavioral tests were performed between 9:00 am and 4:00 pm. Standard food and water were available *ad libitum*. All of the procedures were conducted in accordance with the guidelines for the ethical use of conscious animals in pain research published by the International Association for the Study of Pain (Zimmermann, 1983) and were approved by the Institutional Animal Care Committee of the Butantan Institute (CEUAIB, protocol number 1245/14 and 9766020419) according to ARRIVE guidelines.

### Chemicals and Drug Administration

The following chemicals were obtained from the sources indicated. Morphine (morphine sulfate, União Química, Brazil, 1 μg/kg, 1 or 5 mg/kg (subcutaneous route) or 6, 12, or 24 μg/paw (intraplantar route) was dissolved in sterile saline and injected 60 min before the first nociceptive evaluation. PGE2 was purchased from the Sigma Chemical Co. (St. Louis, MO, United States). A stock solution of PGE2 was prepared by dissolving 500 μg PGE2 in 1 mL of 100% ethanol and then diluting it in sterile saline before injection into the rat paw (100 ng/paw). The percentage of ethanol in the solution injected in the hind paw was 0.2% (Zambelli et al., 2014). ERKI, an ERK inhibitor; SB20358, a p38 inhibitor; SP600125, a JNK inhibitor; PD98059, a MEK inhibitor were dissolved in 5% DMSO and administered intrathecally (i.t.) at the dose of 30 μg (Ma et al., 2001). The oligodeoxynucleotides (ODN)-antisense against CREB (Exxtend-Brazil), or its mismatch, was also administered by intrathecal route at the dose of 30 μg, in a volume of 10 μL once daily for 5 days before morphine injection, including the morphine first day, and then every other day until Day 4 (96 h) (dos Santos et al., 2014). The behavioral assessment was conducted on the day following the last injection (96 h). Briefly, for these intrathecal injections, the rats were anesthetized with 2% isoflurane and a 29-gauge needle was inserted in the subarachnoid space on the midline between L4 and L5 vertebrae (Milligan et al., 2003). The animals recovered consciousness approximately 1 min after discontinuing the anesthetic.

### Behavioral Assessment

#### Mechanical Hyperalgesia Evaluation

To assess the nociceptive threshold, the rat paw pressure test was used (Randall and Selitto, 1957) (Ugo Basile, VA, Italy). The test was applied before and for up to 192 h after morphine treatment or PGE2 injection. Testing was blind in regard to group designation. In this test, an increasing force (measured in g) was applied to the hind paw of the rat and interrupted when the animal withdrew the paw. The force necessary to induce this reaction was recorded as the nociceptive threshold. A maximum pressure of 250 g (i.e., cut-off) was established to minimize damage to the paw. To reduce stress, rats were habituated to the testing procedure a day before experimentation.

#### Thermal Hyperalgesia Evaluation

The Hargreaves technique was used for thermal hyperalgesia studies (using the Ugo Basile Model 7370 Plantar Test). Paw withdrawal responses to noxious heat stimuli were measured in rats as described (Hargreaves et al., 1988). To measure response to noxious heat stimuli, each animal was placed in a Plexiglas chamber on a glass plate located above a light box. Radiant heat was applied by focused infrared (IR) beam to the middle of the plantar surface of rat hind paw. When the animal lifted its foot, the IR beam was turned off. The time interval between the start of the IR beam and the foot lift was defined as the paw withdrawal latency. Each trial was repeated at least 3 times at 5-min intervals for each paw. A cut-off time of 30 s was used to prevent paw tissue damage. Blinding method was implemented strictly for the whole behavioral test.

### Biochemical Studies

#### Quantitative Real-Time Reverse Transcription Polymerase Chain Reaction

For this study, spinal cord (L4-L6) of the rats was collected 96 h after the subcutaneous injection of morphine. Saline treated animals were used as controls. After collection, tissues were rapidly homogenized in TRIZOL reagent (Sigma-Aldrich, St. Louis, MO, United States). Total cellular RNA was purified from tissue according to the manufacturer’s instruction and the purity and integrity of total RNA was measured. Reverse transcription was performed with a reverse transcription reaction (Superscript II; Invitrogen, Thermo Fisher Scientific, Waltham, MA, United States). Real-time PCR was performed using primers specific for the rats genes Fos B, MyC, and cJun and the levels of each gene were normalized to the levels of the rat Gapdh gene. Reactions were conducted on the StepOne Plus (Applied Biosystems) using the SYBR-green fluorescence system (Thermo Fisher Scientific, Waltham, MA, United States). The results were analyzed by the method of quantitative relative expression 2−ΔΔ(ΔCt as previously described. Primer pairs for rats Gapdh, Fos B, MyC, and cJun were as follows: Gapdh fwd: 5′-TCGGTGTGAACGGATTTGGC-3′; Gapdh rev: 5′-CCTTCAGGTGAGCCCCAGC-3′; Fos B fwd: 5′-ACGCCGAGTCCTACTCCAG-3′; Fos B rev: 5′-TCTCCT CCTCTTCGGGAGAC-3′; MyC fwd: 5′-GAAGAACAAG ATGATGAGGAA-3′; MyC rev: 5′-GCTGGTGAGTAGAGAC AT-3′; cJun fwd: 5′-CGGCCCCGAAACTTCTG-3′; cJun rev: 5′-GTCGTTTCCATCTTTGCAGTCA-3′.

#### Western Blot Analysis

Samples of spinal cord (L4-L6) were collected and homogenized in a lysis buffer containing a mixture of protease inhibitors and phosphatase inhibitors (Sigma-Aldrich). The protein concentrations of the lysate were determined using a Bradford assay (Bradford, 1976). Protein samples were separated on a SDS-PAGE gel (10% gradient gel; Bio-Rad) and transferred to nitrocellulose membranes (GE Healthcare). The filters were blocked for 120 min with 5% BSA and incubated overnight at 4°C with a primary antibody against phospho-ERK, phospho-JNK, phospho-p38 or non-phosphorylated forms of these proteins (1:1000; Cell Signaling Technology, Danvers, MA, United States). The membranes were then incubated in the appropriate peroxidase-conjugated secondary antibody (1:5000; anti-rabbit, Sigma-Aldrich, St. Louis, MO, United States, cat. numbers A0545 and A8919, respectively) for 120 min at room temperature and developed using enhanced chemiluminescence (Amersham GE Healthcare Bio-Sciences Corp.; Piscataway, NJ, United States). Quantification analysis of the blots was performed using the UVITEC software. Images were used as representative blots. Densitometric data were measured after normalization to total protein (not phosphorylated).

### Statistical Analysis

The results are presented as the mean ± SEM. For Figures 1–4, 6–8, 9B, and Supplementary Figures S1, S2 statistical evaluation of data was conducted using a two-way analysis of variance (ANOVA) with *post hoc* testing by Tukey. One way ANOVA was used to analyze Figures 5, 9B, 10 with *post hoc* testing by Tukey. A value of *P*< 0.05 was considered significant.

**FIGURE 1:**
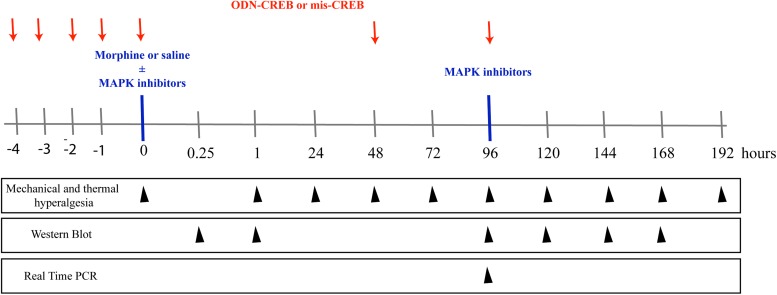
Schematic representation of the experimental design of the work. To assess the mechanical and thermal nociceptive threshold, the rat paw pressure test (Randall and Selitto, 1957), and paw withdrawal responses test to noxious heat stimuli (Hargreaves et al., 1988) were measured, respectively. The tests were applied before and for up to 192 h after morphine treatment (1 μg/Kg, 1 e 5 mg/Kg, s.c. or 6, 12, and 24 μg/paw). The pharmacological inhibitors of MAPKs, ERK inhibitor (ERKI), p38 inhibitor (SB20358) or JNK inhibitor (SP660125), were injected by intrathecal route (i.t), and were administrated concomitantly to morphine or 95 h after systemic morphine. The nociceptive thresholds were evaluated before and for up to 168 h after morphine treatment. The expression and phosphorylation of ERK, p38, JNK, and CREB were evaluated by Western Blot at 0.25, 1, 96, 120, 144, and 168 h after morphine treatment (5 mg/Kg). The intrathecal administrations of the antisense-ODN against CREB, or a mismatch sequence, were performed for 5 days before morphine administration (including the day of morphine injection) and every other day afterward. The nociceptive thresholds were evaluated before and 1, 48, 72, and 96 h after morphine treatment (5 mg/Kg). The mRNA expression of MYC, FOS B, and cJUN were evaluated by Real- Time PCR at 96 h after morphine treatment (5 mg/Kg).

**FIGURE 2:**
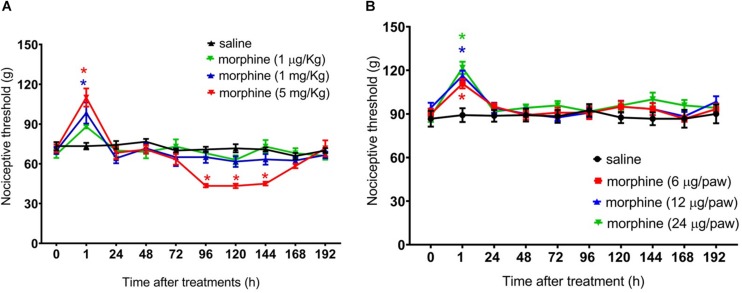
Effect of morphine in the rat mechanical nociceptive threshold. Nociceptive threshold was obtained in the rat paw pressure test, before (0) and 1, 24, 48, 72, 96, 120, 144, and 192 h after subcutaneous injection of morphine (1 μg/Kg, 1 mg/Kg, and 5 mg/Kg) **(A)** and intraplantar injection of morphine (6 μg/paw, 12 μg/paw, and 24 μg/paw) **(B)**. Morphine was injected in naïve rats. Data represent mean values ± SEM for six rats per group. ^∗^ significantly different from baseline (0). Data were analyzed by two-way analysis of variance (ANOVA) with *post hoc* testing by Tukey.

**FIGURE 3:**
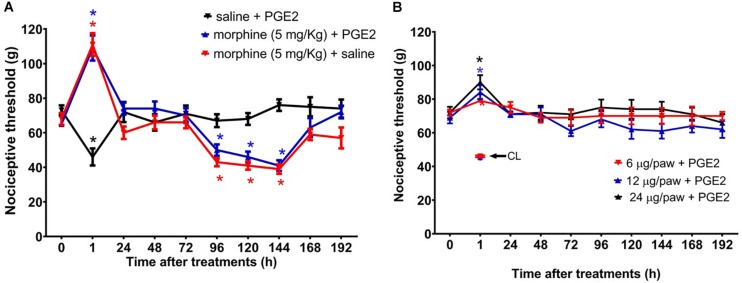
Effect of morphine in the mechanical nociceptive threshold of rats treated with prostaglandin E2 (PGE2). Nociceptive threshold was obtained in the rat paw pressure test, before (0) and 1, 24, 48, 72, 96, 120, 144, and 192 h after subcutaneous injection of morphine (5 mg/Kg) **(A)** and intraplantar injection of morphine (6 μg/paw, 12 μg/paw, and 24 μg/paw) **(B)**. PGE2 (100 ng/paw) was injected 2 h before the opioid administration. The contralateral paw threshold (CL) is indicated with an arrow. Data represent mean values ± SEM for six rats per group. ^∗^ significantly different from baseline (0). Data were analyzed by two-way analysis of variance (ANOVA) with *post hoc* testing by Tukey.

**FIGURE 4:**
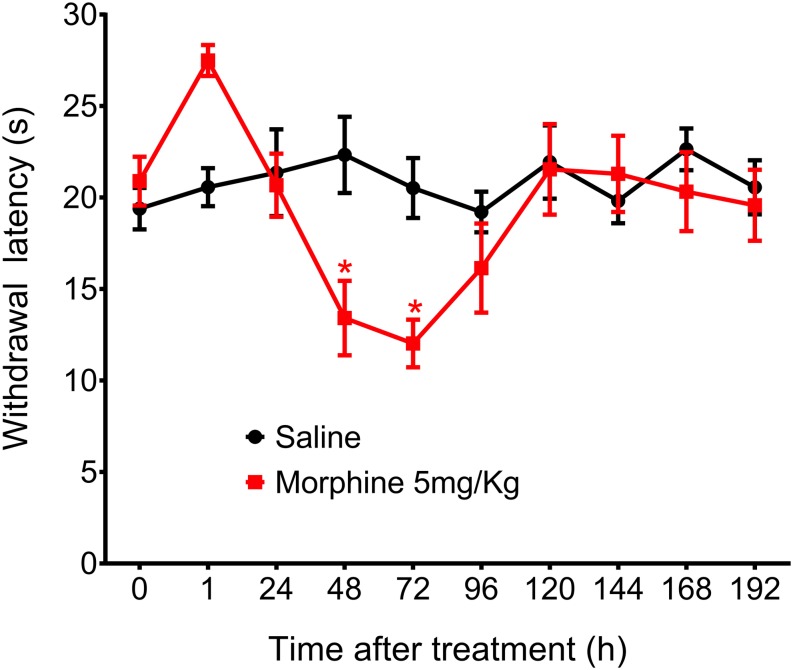
Effect of morphine in the rat thermal nociceptive threshold. Thermal sensitivity, as assessed by hind paw withdrawal threshold, was measured by the Hargreaves method, before (0) and 1, 24, 48, 72, 96, 120, 144, and 192 h after subcutaneous injection of morphine (5 mg/Kg). Data represent mean values ± SEM for six rats per group. ^∗^ significantly different from baseline (0). Data were analyzed by two-way analysis of variance (ANOVA) with post hocpost-hoc testing by Tukey.

**FIGURE 5:**
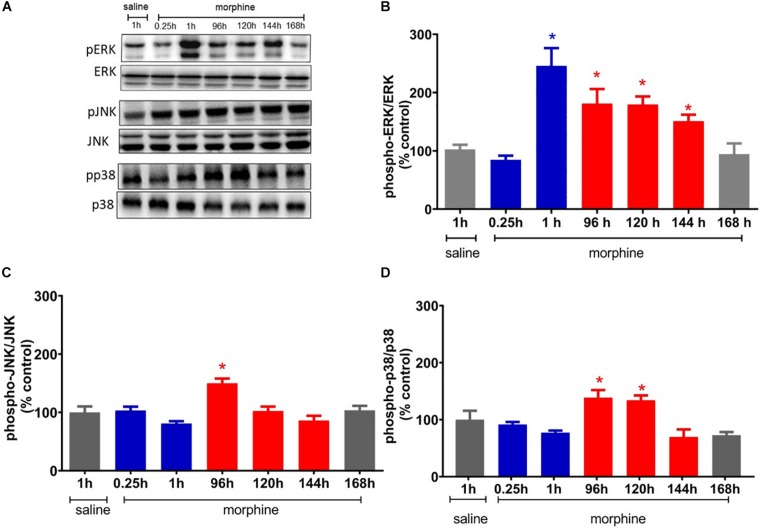
Morphine systemic injection effect on protein expression of ERK1/2 **(A)**, JNK **(B)**, and p38 **(C)** MAPKs. The changes in protein levels of MAPKs were determined by immunobloting, in lumbar spinal cord obtained 0.25, 1, 24, 48, 72, 120, 144, and 192 h after morphine injection (5 mg/Kg, s.c.). **(D)** Representative blots showing the phosphorylated and total ERK1/2, JNK, and p38 levels in the total lisate of spinal cord. Data are presented as mean ± SEM and expressed as % of control (saline) animals. ^∗^ significantly different from mean values of saline treated animals, *n* = 6 per group (*p* = 0.05). Data were analyzed by one-way analysis of variance (ANOVA) with post hoc testing by Tukey.

Because we and others have previously demonstrated that peripheral sensitization may interfere with the analgesic efficacy of opioids (Obara et al., 2009; Zambelli et al., 2014), we next sought to investigate whether a peripheral sensitization would exacerbate and/or extend the morphine-induced hyperalgesia. To address this question, rats received an intraplantar injection of 100 ng/paw PGE2 that caused a significant decrease in the nociceptive threshold, with a peak response at 3 h, when compared with the basal values. The systemic morphine (5 mg/Kg) blocks PGE2-induced hyperalgesia and induces delayed hyperalgesia, starting at 96 h and lasting until 144 h after morphine (Figure 3A). We also checked whether the effect of peripherally administered morphine alters PGE2-induced hyperalgesia. As detected in the absence of sensitization, the peripheral morphine injection causes analgesia, without generating hyperalgesia (Figure 3B). Intraplantar injection of morphine did not modify the PGE2-induced hyperalgesia in the contralateral paw, indicating again that the opioid, at doses presently used exerts only a local (peripheral) effect. Importantly, these effects were only observed in the paw injected with morphine, excluding a systemic effect. Taken together, these results suggest that peripheral sensitization (by PGE2) neither interferes with delayed hyperalgesia after systemic morphine injection nor with the absence of hyperalgesia when morphine was peripherally injected.

### Experimental Design

The schematic Figure 1 summarizes the experimental design of this work.

## Results

First, we performed a dose response curve of systemically injected morphine on the nociceptive threshold of rats. The mechanical threshold was assessed 1 h after morphine and every day for 8 days (192 h). A single injection of morphine at 1 mg/Kg or 5 mg/Kg, s.c., induces significant increase in the mechanical nociceptive threshold (analgesia), at 1 h after the administration as compared to the baseline. Importantly, rats treated with morphine (5 mg/Kg), besides displaying analgesia, also display a long-lasting decrease in the nociceptive threshold (hyperalgesia), starting at Day 4 (96 h) and lasting until Day 6 (144 h). A lower dose of morphine (1 μg/Kg, s.c.) neither induces analgesia nor hyperalgesia (Figure 2A). As expected, no difference in the nociceptive threshold was detected in control rats treated with saline. Importantly, morphine did not affect rat gross motor skills. No differences were detected in the mean number of squares crossed in an open field or in rearing behavior between morphine and saline treated rats (data not shown). Next, we evaluated the effect of peripherally injected morphine on the mechanical nociceptive threshold. The intraplantar injection of morphine, at the doses of 6, 12, or 24 μg/paw, increases paw nociceptive threshold in the ipsilateral paw, without inducing hyperalgesia (Figure 2B). Also, intraplantar administration of morphine did not interfere with the nociceptive threshold of the contralateral paw (data not shown), indicating that, at these doses, and the opioid induces only local (peripheral) effect. Taken together, the results obtained for the systemic and intraplantar administration of morphine indicate that (a) a single systemic injection of morphine can induce a biphasic response in the nociceptive threshold of uninjured rats (early analgesia and long lasting delayed hyperalgesia) and (b) central mechanisms are involved in morphine-induced delayed hyperalgesia.

In addition to mechanical hyperalgesia, we also investigated whether a single morphine injection (5 mg/Kg) would induce thermal hyperalgesia, The results showed that morphine, in the same dose that induces mechanical delayed hyperalgesia also induces thermal hyperalgesia. However, thermal hyperalgesia started 48 h after morphine and lasted for just 24 h (Figure 4). Because morphine did not induce significant analgesia in this hyperalgesia model and because thermal hyperalgesia lasted only 24 h, we decided to conduct the further experiments using only the mechanical nociceptive stimulus.

### Spinal ERK Activation Correlates With Analgesia and Hyperalgesia, While p38 and JNK Activation Correlate With Morphine-Induced Hyperalgesia

To elucidate the potential mechanisms involved in the mechanical hyperalgesia induced by a single injection of morphine, we first investigated whether ERK, p38, and JNK expression and phosphorylation were altered by morphine. The systemic injection of a single morphine dose (5 mg/Kg) increases the level of phosphorylated ERK1/2 in lumbar spinal cord, 1 h after injection, when compared to saline treated rats. ERK1/2 activation is also detected at 96, 120, and 144 h. The morphine injection also increases the levels of JNK phosphorylation at 96 h after injection and of p38 at 96 and 120 h. Taken together, these data suggest that ERK1/2 is activated in the period in which morphine is inducing both analgesia and hyperalgesia. However, p38 and JNK are activated only in the period that hyperalgesia is detected (Figure 5).

### Spinal MAPKs Are Involved in Both Analgesia and Hyperalgesia Induced by a Single Dose of Systemic Morphine

In order to test the functional significance of the increased MAPK activation in the spinal cord, pharmacological inhibitors of MAPKs were injected by intrathecal route. ERK inhibitor (ERK-I), p38 inhibitor (p38-I, SB20358) or JNK inhibitor (JNK-I, SP660125) were administered in two experimental conditions (a) concomitantly with morphine or (b) 95 h after systemic morphine. We chose these two time points because they correspond to the analgesic and hyperalgesic effects, respectively. Importantly, because the inhibition of MEK, an up-stream MAPK kinase, interferes with PGE2 induced hyperalgesia, the following experiments were performed in naïve rats, i.e., without sensitization (Supplementary Figure S1).

**FIGURE 6:**
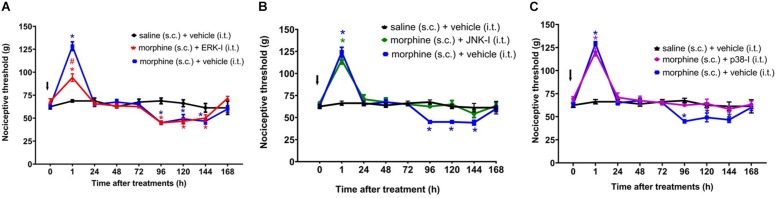
Effect of MAPK inhibitors on morphine induced analgesia and hyperalgesia. Nociceptive threshold was obtained in the rat paw pressure test, before (0) and 1, 24, 48, 72, 96, 120, 144, and 192 h after subcutaneous injection of morphine (5 mg/Kg). The ERK inhibitor (ERK-I) **(A)**, JNK inhibitor (JNK-I, SP660125) **(B)**, and p38 inhibitor (p38-I, SB20358) **(C)** were injected by intrathecal route (30 μg/30 μL), concomitantly to morphine (*t* = 0). The arrows indicate the time of inhibitors administration. Data represent mean values ± SEM for six rats per group. ^∗^ significantly different from baseline (0). ^#^ significantly different from morphine (s.c.) + vehicle (i.t.). Data were analyzed by two-way analysis of variance (ANOVA) with *post hoc* testing by Tukey.

The ERKI, injected concomitantly to morphine, partially decreases morphine-induced analgesia without interfering with morphine-induced hyperalgesia (Figure 6A). In contrast, p38-I and JNK-I did not interfere with morphine-induced analgesia but prevented the development of hyperalgesia (Figures 6B,C).

**FIGURE 7:**
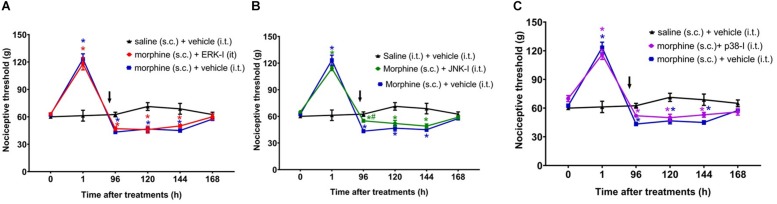
Effect of MAPK inhibitors on morphine-induced hyperalgesia. Nociceptive threshold was obtained in the rat paw pressure test, before (0) and 1, 24, 48, 72, 96, 120, 144, and 192 h after subcutaneous injection of morphine (5 mg/Kg). The ERK inhibitor (ERK-I) **(A)**, JNK inhibitor (JNK-I, SP660125) **(B)**, and p38 inhibitor (p38-I, SB20358) **(C)** were injected by intrathecal route (30 μg/30 μL), concomitantly 96 h after morphine (*t* = 96 h). The arrows indicate the time of inhibitors administration. Data represent mean values ± SEM for six rats per group. ^∗^ significantly different from baseline (0). ^#^ significantly different from morphine (s.c.) + vehicle (i.t.). Data were analyzed by two-way analysis of variance (ANOVA) with *post hoc* testing by Tukey.

The next step was to evaluate whether inhibiting ERK1/2, p38 and JNK at 95 h and 30 min after morphine, i.e., 30 min before the assessment of hyperalgesia, is able to prevent this condition. The ERK or p38 inhibition did not interfere the morphine-induced hyperalgesia (Figure 7). However, JNK inhibition partially reverts the opioid hyperalgesic effect. Control animals injected only with the inhibitors, did not display changes in the nociceptive threshold (Supplementary Figure S2). Together, these data indicate that p38 and JNK contribute to the initiation of hyperalgesia after a single injection of systemic morphine. Interestingly, once the signaling is triggered, the pharmacological inhibition of p38 and JNK can no longer completely restores the nociceptive threshold.

**FIGURE 8:**
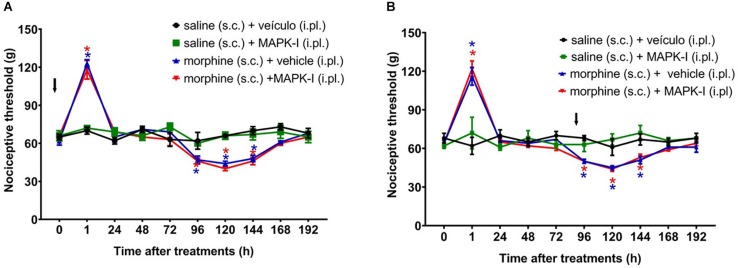
Effect of peripheral MAPK on morphine-induced analgesia and hyperalgesia. Nociceptive threshold was obtained in the rat paw pressure test, before (0) and 1, 24, 48, 72, 96, 120, 144, and 192 h after subcutaneous injection of morphine (5 mg/Kg). The ERK inhibitor (ERK-I), JNK inhibitor (JNK-I, SP660125) or p38 inhibitor (p38-I, SB20358) were injected, by intraplantar route **(A)** concomitantly to morphine, **(B)** 96 h after morphine (*t* = 96 h). Data represent mean values ± SEM for six rats per group. ^∗^ significantly different from baseline (0). The arrows indicate the time of inhibitors administration. Data were analyzed by two-way analysis of variance (ANOVA) with *post hoc* testing by Tukey.

We next asked whether the peripheral MAPKs participate in the morphine central effect. In order to address this question, the MEK inhibitor (PD98059) was injected by intraplantar route. The inhibitor did not interfere with morphine-induced hyperalgesia when injected concomitantly with morphine or 95 h afterward (Figure 8). Our data suggest that only central MAPKs are involved in the hyperalgesia induced by a single injection of morphine.

### Downregulation of cAMP-Responsive Element Binding Protein (CREB) Expression Impairs Morphine-Induced Hyperalgesia

**FIGURE 9:**
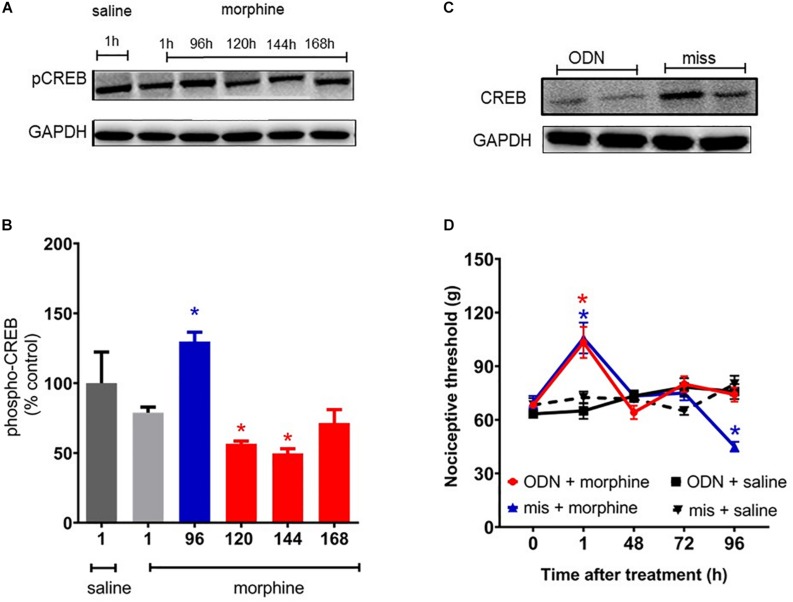
cAMP-responsive element binding protein (CREB) activation mediates morphine-induced delayed hyperalgesia. The changes in phosphorylated protein levels of CREB were determined by immunobloting, in lumbar spinal cord obtained 1, 96, 120, 144, and 168 h after morphine injection (5 mg/Kg, s.c.). **(A)** Representative blots **(B)** percentage changes in the phosphorylated CREB levels in the total lysate of lumbar spinal cord compared to saline treated animals. The ODN antisense or mismatch (30 μg/10 μL) were injected by intrathecal route 5 days prior morphine and every other day afterward. **(C)** Representative blots of CREB expression after injection of ODN antisense or mismatch. **(D)** Nociceptive threshold was obtained in the rat paw pressure test, before (0) and 1, 24, 48, 72, and 96 h after subcutaneous injection of morphine (5 mg/Kg, s.c.) in the presence of ODN antisense or mismatch. Data are presented as mean ± SEM and expressed as % of control (saline) animals. ^∗^ significantly different from mean values of saline treated animals, *n* = 6 per group (*p* < 0.05). Data were analyzed by one-way **(B)** and **(D)** two-way analysis of variance (ANOVA) with *post hoc* testing by Tukey.

It has been extensively reported that MAPK signaling culminates in activating CREB, a key regulator of gene expression. Therefore, we evaluated the CREB phosphorylation levels in spinal cord of the animals. CREB phosphorylation levels are elevated 96 h after morphine, a period that overlaps with the hyperalgesic effect (Figure 9A). In order to confirm that the CREB activation has an important function in hyperalgesia, a functional knockdown of CREB was achieved by intrathecal administrations of the antisense-ODN against CREB, or a mismatch sequence. Antisense injections were performed for 5 days before morphine administration (including the day of morphine injection) and every other day afterward (48 and 96 h). As shown in Figure 9D, hyperalgesia induced by a single injection of morphine was completely abolished by the antisense-ODN against the CREB. These data suggest that CREB activation plays a critical role in morphine-induced hyperalgesia. The decrease in phosphorylated CREB expression was confirmed by Western blot.

### A Single Injection of Morphine Is Sufficient to Up-Regulates Transcriptional Factors Related to MAPK-CREB Pathway

**FIGURE 10:**
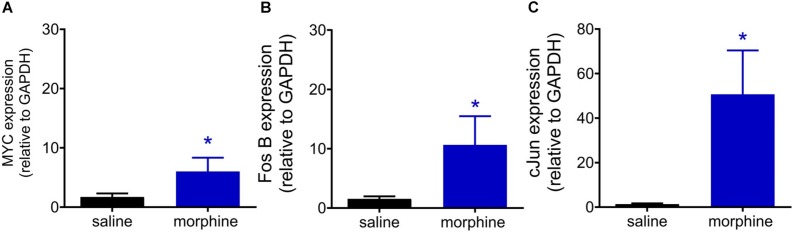
Morphine systemic injection effect on gene expression of MYC **(A)**, Fos B **(B)**, and cJUN **(C)** transcriptional factors. The changes in mRNA levels of immediate early genes were determined by real time PCR, in dorsal root ganglia (DRG) obtained 96 h after morphine injection (5 mg/Kg). Data are presented as mean of 6 animals ± SEM and expressed as % of control (saline treated) animals. ^∗^ significantly different from mean values of naïve animals (*p* < 0.05). Data were analyzed by one-way analysis of variance (ANOVA) with *post hoc* testing by Tukey.

Phosphorylated CREB is able to bind to the calcium/cAMP response elements (CalCRE) in the promoter regions of the genes, such as immediate early genes. Then, we performed an exploratory study evaluating the mRNA expression of transcription factors involved MAPK-CREB signaling that may regulate the morphine-induced hyperalgesia. This experiment was performed in samples from spinal cord collected 96 h after morphine, the time point when the hyperalgesia is first detected. Morphine injection up regulates the mRNA expression of MYC (2.5 fold), FOS B (5.6 fold), and cJUN (37.6 fold) (Figure 10). Together, these data reinforce the functional importance of MAPK in morphine-induced signaling and provide evidences that morphine can be compared to a stress-related stimulus.

## Discussion

Our data revealed that a single injection of morphine induces an early analgesic effect characterized by an increase in the nociceptive threshold, followed by a delayed hyperalgesia, i.e., a long-lasting decrease in the nociceptive threshold. Moreover, we provided behavioral and biochemical evidences that the MAPK signaling pathway, especially JNK and p38, and CREB activation are critical for the opioid-induced long-lasting hyperalgesia.

In our study, we first evaluated the subcutaneous morphine effects on the mechanical threshold of rats. Morphine at 5 mg/Kg induces an early analgesia (1 h after injection) followed by mechanical hyperalgesia that begins 4 days after injection and lasts for 2 days. We also showed that rats do not develop delayed hyperalgesia when morphine is locally (i.pl.) administered. Importantly, the doses used in the i.pl. injections displayed a local effect because anti-nociception was only detected in the paw injected with the drug and not in the contralateral paw. These results could suggest that a central morphine action would be required to the delayed hyperalgesia development. However, morphine at 5 mg/Kg also induces thermal hyperalgesia, which started earlier as compared to the mechanical hyperalgesia. We do not have experimental data to explain the time separation between thermal and mechanical hyperalgesia. However, electrophysiological studies have subdivided Aδ nociceptors into two main classes (Basbaum et al., 2009). First, type I, that respond to both mechanical and chemical stimuli and, type II Aδ, that have a much lower heat threshold but a very high mechanical threshold and is responsible to mediate the first acute pain response to noxious heat. Then, is possible that in our experimental conditions, distinctive nociceptive afferents can be activated by thermal and/or mechanical stimuli and induce different hyperalgesic responses, in addition to the central site of action. Another hypothesis for the differences detected between thermal and mechanical responses is that while Randall Selitto test applies an increasing force to determine the mechanical nociceptive threshold, the Hargreaves thermal test evaluates the nociceptive reaction latencies in response to a thermal aversive stimulus. Therefore, there is a limitation in our study since we did not determine the thermal nociceptive threshold, which makes challenging the comparison between these two tests. Methods that slowly increase thermal stimulus would be beneficial to make a better comparison with the paw pressure test (Yalcin et al., 2009; Alshahrani et al., 2012). Further studies are needed to address this interesting issue.

To our knowledge, the present study is the first one to demonstrate that a single dose of systemic morphine is sufficient to induce mechanical and thermal hyperalgesia. Importantly, the development of delayed hyperalgesia was unaffected by PGE2 injection, suggesting that morphine hyperalgesic effect does not depend or are altered by sensitization of neuron peripheral terminals (Ferreira and Lorenzetti, 1981; Zambelli et al., 2014). On the other hand, the magnitude of morphine-induced analgesia in PGE2 sensitized rats was the same as for naïve (non-sensitized) rats. Clinical and experimental results have shown that the peripheral analgesic efficacy of opioids is enhanced in the presence of inflammation and tissue injury (Stein, 1993, Obara et al., 2009, Stein and Lang, 2009). Our group previously demonstrated that the PGE2 intraplantar injection increases the activation of the μ- and κ-opioid receptors by their corresponding agonist, enhancing the agonist‘s analgesic effect. We showed that PGE2 up-regulates the peripheral protein expression levels of the mu- and kappa-opioid receptors and activates specific intracellular signaling pathways (MAPKs and PKCζ) that may contribute to the analgesic effect (Zambelli et al., 2014). Therefore, despite the marked hyperalgesia induced by PGE2, this eicosanoid enhances the morphine efficacy, without interfering with the hyperalgesia development.

It is important to mention that the analgesia produced by intraplantar morphine was not dose-dependent. In our study, the mechanical nociceptive testing was applied on the dorsal surface of the hind paw and the injections were performed on the plantar surface. Therefore, we cannot rule out the possibility that this effect may be related to testing at a site distal from the site of administration of morphine. Further studies are necessary to address whether different sites of injection and nociceptive evaluation interfere with the pain threshold recording. However, there are reports showing that the paw pressure test, applied to the dorsal surface of the rat paw, is as sensitive as the electronic von Frey applied to the injection site (plantar surface). Both methods detected time vs. treatment and dose vs. time interactions in prostaglandin E2 and carrageenan-induced hypernociception, indicating that the site of stimulus application (dorsal or plantar side of the paw) may not interfere with the detection of dose-response curves (Vivancos et al., 2004).

Several studies have reported a progressive reduction of baseline thresholds after daily systemic opioid injections, during 10 days (Corder et al., 2017), or after opioid escalating doses (Shimoyama et al., 1996; Corder et al., 2017), or by continuous infusion these drugs for 7 days (Horvath et al., 2010). Despite these evidences, few studies monitored the nociceptive threshold for several days after an acute morphine injection. In this respect, Van Elstraete et al. (2005) showed that a single intrathecal injection of morphine induces short time analgesia, followed by a long-lasting (48 h) hyperalgesia (Van Elstraete et al., 2005). In this study, delayed hyperalgesia after an equivalent single dose of systemic morphine was significantly less during the first 2 days after morphine administration. Similarly, our data shows that spinal mechanisms are involved in the morphine hyperalgesic effect after a single injection of morphine. It is important to mention that recent studies reported that repeated systemic injection of morphine induces hyperalgesia mediated by peripheral mu-opioid receptors (Corder et al., 2017), suggesting that distinct mechanisms may be involved in the delayed hyperalgesia detected in our study. However, there is strong evidence showing that a single systemic morphine injection sensitize central and peripheral terminals. In fact, Ferrari et al. (2019) demonstrated that systemic morphine prolongs the mechanical hyperalgesia produced by PGE2, when evaluated 5 days after morphine injection. Therefore, despite the absence of hyperalgesia after morphine intraplantar injection, the peripheral nociceptors may be sensitized and much more susceptible to chronic hyperalgesia development after an insult.

The fact that morphine induces an adaptive process at molecular, synaptic, cellular and circuit levels (Rivat and Ballantyne, 2016) suggests that morphine-induced hyperalgesia and pathological pain may share common cellular mechanisms (Christie, 2008). Studies have shown that ERK1/2, JNK, and p38 are involved with the effects of chronic morphine exposure and that quinases modulate morphine-induced analgesic tolerance. Intrathecal administration of the selective p38, ERK, or JNK inhibitors significantly reduced morphine-induced tolerance and associated thermal hyperalgesia by suppressing the morphine-induced increase of TRPV1 expression (Chen et al., 2008). However, the role of MAPKs after a single systemic injection of morphine in the nociceptive threshold is unknown. Our biochemical results indicate that the levels of phosphorylated ERK1/2 in spinal cord are elevated in periods that correlate with morphine-induced analgesia (1 h) and delayed hyperalgesia (96 h). On the other hand, the increased phosphorylation levels of p38 and JNK only correlate with the delayed hyperalgesia. The fact that we did not detect increased JNK phosphorylation at 120 and 144 h and p38 phosphorylation at 144 h does not exclude their participation in morphine-induced hyperalgesia. MAPKs can be transiently phosphorylated and dephosphorylated, activating downstream signaling pathways that may be involved in the long-lasting biological effects. Interestingly, our functional studies showed that the i.t. injection of p38 and JNK inhibitors, but not ERK1/2 inhibitor, abrogate the morphine-induced delayed hyperalgesia. The analgesic effect was partially decreased by the ERK1/2 inhibitor, but not by p38 and JNK inhibitors. Here, we did not examine the levels of ERK, p38, and JNK after the pharmacological inhibition, but the efficacy of these drugs have been extensively reported (Qu et al., 2016). In this regard, the i.t. injection of these inhibitors not only diminished protein expression of p38, ERK, and JNK in the pain pathways but also affected the level of gene expressions (Qu et al., 2016). We should mention that the doses of the inhibitors used in this study were chosen based on their optimal effects as observed in previous dose-response studies (Wang et al., 2009; Deng et al., 2018).

Taken together, the behavioral and biochemical data provide strong evidence that a single s.c. morphine injection induces a biphasic response: analgesia followed by a delayed hyperalgesia mediated, at least in part, by spinal ERK1/2 and p38/JNK activation, respectively. Importantly, despite the increased ERK1/2 phosphorylation levels, the ERK inhibition is not sufficient to prevent the delayed hyperalgesia. Therefore, our data suggest that morphine sensitizes the spinal cord through p38 and JNK leading to an reduction in the mechanical threshold and consequently onset of hyperalgesia. Despite the fact that ERK1/2 plays a minor role in our model, we do not exclude the role of this kinase in morphine effects. Several reports demonstrate that ERK1/2 mediates morphine-induced tolerance (Ma et al., 2001; Sanna et al., 2014), and the present findings do not exclude the possibility that spinal ERK1/2 activation by prolonged systemic or intrathecal morphine can contribute to the decrease of nociceptive thresholds. It is important to stress that our experiments were performed using total protein extracts from lumbar spinal cord, so, identification of cell types that mediate MAPK activation needs to be determined by immunofluorescence studies.

Mitogen-activated protein kinases are fundamental players for the onset and maintenance of pain. Their activation produce intracellular responses by changing transcription as well as by posttranslational and translational modification of target proteins. Phosphorylated CREB binds to the calcium/cAMP response elements (CalCRE) in the promoter regions of a number of immediate early genes (c-Jun, Jun-B, Jun-D, c-Myc, c-Fos, and Fos B) and neuropeptide genes (Kanjhan, 1995). In our study, a single morphine injection increased CREB phosphorylation in the spinal cord, in a period of time that correlates to the mechanical hyperalgesia initiation. Our functional studies using antisense to knockdown the CREB expression showed that this protein is critical for the delayed hyperalgesia development. Despite our results, the role of JNK and p38 in CREB phosphorylation remain to be elucidated; however, we speculate that a single injection of systemic morphine induces hyperalgesia by activating p38 and JNK that, in turn, activates CREB. Studies have demonstrated that chronic morphine administration induces an increase in CREB phosphorylation and neuropeptide release mediated by ERK1/2 and p38, with JNK kinase pathways playing a lesser role in these effects (Ma et al., 2001; Wang et al., 2009).

The immediate-early genes encode transcription factors that function to regulate the expression of other so-called late response genes and therefore can trigger a cascade of events that may lead to functional and morphological alterations of neuronal and non-neuronal cells. Our exploratory study demonstrates that the early genes such as c-Myc, Fos-B, and c-Jun are upregulated 96 h after morphine injection, with c-Jun being increased more than thirty times. Of note, c-Jun responds to stress-related stimuli and is the major transcription factor downstream JNK. These data reinforce our behavioral data showing that a single injection of morphine is able to induce neuropathological changes in the spinal cord. The activation of JNK and p38 induced by morphine may participate in the delayed hyperalgesia by regulating the downstream targets, such as calcitonin gene-related peptide, substance P, nitric oxide, transient receptor potential vanilloid 1, and proinflammatory cytokines.

Taken together, our findings show that a single analgesic dose of morphine results in activation of a pronociceptive system as demonstrated by the reduction of nociceptive thresholds through the activation the MAPK-CREB signaling. The fact that a short-term administration of an opioid can enhance hyperalgesia may be of clinical importance due to the possibility of analgesia counteraction and/or paradoxical enhancement of a pre-existing pain condition during opioid therapy.

## Data Availability Statement

The raw data supporting the conclusions of this manuscript will be made available by the authors, without undue reservation, to any qualified researcher.

## Ethics Statement

The animal study was reviewed and approved by the Animal Care Committee of the Butantan Institute.

## Author Contributions

YC and VZ conceived and designed this study. VZ wrote the manuscript with input of YC, GP, and FC. LP and FC performed the behavioral experiments. BF and NH performed the Western blot. FC and BF performed the PCR. GP, YC, BF, LP, and VZ analyzed the data.

## Conflict of Interest

The authors declare that the research was conducted in the absence of any commercial or financial relationships that could be construed as a potential conflict of interest.

## Supplementary Material

FIGURE S1 Effect of MAPK inhibition on prostaglandin E2-induced hyperalgesia. Nociceptive threshold was obtained in the rat paw pressure test, before (0) and 3, 4, and 6 h after intraplantar PGE2 injection (100 ng/paw). MEK inhibitor (PD 9805) was injected in the paw (30 μg/paw). Data represent mean values ± SEM for six rats per group. ^∗^ significantly different from baseline (0). Data were analyzed by two-way analysis of variance (ANOVA) with *post hoc* testing by Tukey.

FIGURE S2 Effect of MAPK inhibitors *per se* on the rat nociceptive threshold. Nociceptive threshold was obtained in the rat paw pressure test, before (0) and 1, 3, and 24 h after subcutaneous intrathecal injection of ERK inhibitor (ERK-I), JNK inhibitor (JNK-I, SP660125), or p38 inhibitor (p38-I, SB20358). Data represent mean values ± SEM for six rats per group. ^∗^ significantly different from baseline (0). Data were analyzed by two-way analysis of variance (ANOVA) with *post hoc* testing by Tukey.

## References

[B1] Alshahrani S. Fernandez-Conti F. Araujo A. DiFulvio M. (2012). Rapid determination of the thermal nociceptive threshold in diabetic rats. *J. Vis. Exp.* 63:e3785. 10.3791/3785 22643870 PMC3466937

[B2] Angst M. S. Clark J. D. (2006). Opioid-induced hyperalgesia: a qualitative systematic review. *Anesthesiology* 104 570–587. 10.1097/00000542-200603000-00025 16508405

[B3] Angst M. S. Koppert W. Pahl I. Clark D. J. Schmelz M. (2003). Short-term infusion of the μ-opioid agonist remifentanil in humans causes hyperalgesia during withdrawal. *Pain* 106 49–57. 10.1016/S0304-3959(03)00276-8 14581110

[B4] Basbaum A. I. Bautista D. M. Scherrer G. Julius D. (2009). Cellular and molecular mechanisms of pain. *Cell* 139 267–284. 10.1016/j.cell.2009.09.028 19837031 PMC2852643

[B5] Bradford M. (1976). A rapid and sensitive method for the quantitation of microgram quantities of protein utilizing the principle of protein-dye binding. *Anal. Biochem.* 72 248–254. 10.1006/abio.1976.9999 942051

[B6] Chen Y. Geis C. Sommer C. (2008). Activation of TRPV1 contributes to morphine tolerance: involvement of the mitogen-activated protein kinase signaling pathway. *J. Neurosci.* 28 5836–5845. 10.1523/JNEUROSCI.4170-07.2008 18509045 PMC6670790

[B7] Chen Y. Sommer C. (2009). The role of mitogen-activated protein kinase (MAPK) in morphine tolerance and dependence. *Mol. Neurobiol.* 40 101–107. 10.1007/s12035-009-8074-z 19468867

[B8] Christie M. J. (2008). Cellular neuroadaptations to chronic opioids: tolerance, withdrawal and addiction. *Br. J. Pharmacol.* 154 384–396. 10.1038/bjp.2008.100 18414400 PMC2442443

[B9] Corder G. Tawfik V. L. Wang D. Sypek E. I. Low S. A. Dickinson J. R. (2017). Loss of μ opioid receptor signaling in nociceptors, but not microglia, abrogates morphine tolerance without disrupting analgesia. *Nat. Med.* 23 164–173. 10.1038/nm.4262 28092666 PMC5296291

[B10] Deng M. Chen S. Chen H. Luo Y. Dong Y. Pan H. (2018). Mitogen-activated protein kinase signaling mediates opioid-induced presynaptic NMDA receptor activation and analgesic tolerance. *J. Neurochem.* 148:jnc.14628. 10.1111/jnc.14628 30444263 PMC6340739

[B11] dos Santos G. G. Dias E. V. Teixeira J. M. Athie M. C. P. Bonet I. J. M. Tambeli C. H. (2014). The analgesic effect of dipyrone in peripheral tissue involves two different mechanisms: neuronal KATP channel opening and CB1 receptor activation. *Eur. J. Pharmacol.* 741 124–131. 10.1016/j.ejphar.2014.07.019 25058903

[B12] Ferrari L. F. Araldi D. Bogen O. Green P. G. Levine J. D. (2019). Systemic morphine produces dose-dependent nociceptor-mediated biphasic changes in nociceptive threshold and neuroplasticity. *Neuroscience* 398 64–75. 10.1016/j.neuroscience.2018.11.051 30529265 PMC9948647

[B13] Ferreira S. H. Lorenzetti B. B. (1981). Prostaglandin hyperalgesia, IV: a metabolic process. *Prostaglandins* 21 789–792. 10.1016/0090-6980(81)90235-5 6280243

[B14] Hargreaves K. Dubner R. Brown F. Flores C. Joris J. (1988). A new and sensitive method for measuring thermal nociception in cutaneous hyperalgesia. *Pain* 32 77–88. 10.1016/0304-3959(88)90026-7 3340425

[B15] Horvath R. J. Landry R. P. Romero-Sandoval E. A. DeLeo J. A. (2010). Morphine tolerance attenuates the resolution of postoperative pain and enhances spinal microglial p38 and extracellular receptor kinase phosphorylation. *Neuroscience* 169 843–854. 10.1016/J.NEUROSCIENCE.2010.05.030 20493931 PMC2904400

[B16] Ji R.-R. Gereau R. W. Malcangio M. Strichartz G. R. (2009). MAP kinase and pain. *Brain Res. Rev.* 60 135–148. 10.1016/j.brainresrev.2008.12.011 19150373 PMC2666786

[B17] Ji R.-R. Strichartz G. (2004). Cell signaling and the genesis of neuropathic pain. *Sci. STKE* 2004:reE14. 10.1126/stke.2522004re14 15454629

[B18] Ji R.-R. Woolf C. J. (2001). Neuronal plasticity and signal transduction in nociceptive neurons: implications for the initiation and maintenance of pathological pain. *Neurobiol. Dis.* 8 1–10. 10.1006/nbdi.2000.0360 11162235

[B19] Johnson G. L. Lapadat R. (2002). Mitogen-activated protein kinase pathways mediated by ERK, JNK, and p38 protein kinases. *Science* 298 1911–1912. 10.1126/science.1072682 12471242

[B20] Kanjhan R. (1995). Opioids and pain. *Clin. Exp. Pharmacol. Physiol.* 22 397–403. 10.1111/j.1440-1681.1995.tb02029.x 8582088

[B21] Lee M. Silverman S. M. Hansen H. Patel V. B. Manchikanti L. (2011). A comprehensive review of opioid-induced hyperalgesia. *Pain Physician* 14 145–161. 21412369

[B22] Ma W. Zheng W. H. Powell K. Jhamandas K. Quirion R. (2001). Chronic morphine exposure increases the phosphorylation of MAP kinases and the transcription factor CREB in dorsal root ganglion neurons: an in vitro and in vivo study. *Eur. J. Neurosci.* 14 1091–1104. 10.1046/j.0953-816x.2001.01731.x 11683901

[B23] Milligan E. D. Twining C. Chacur M. Biedenkapp J. O’Connor K. Poole S. (2003). Spinal glia and proinflammatory cytokines mediate mirror-image neuropathic pain in rats. *J. Neurosci.* 23 1026–1040. 10.1523/jneurosci.23-03-01026.2003 12574433 PMC6741915

[B24] Obara I. Parkitna J. R. Korostynski M. Makuch W. Kaminska D. Przewlocka B. (2009). Local peripheral opioid effects and expression of opioid genes in the spinal cord and dorsal root ganglia in neuropathic and inflammatory pain. *Pain* 141 283–291. 10.1016/j.pain.2008.12.006 19147290

[B25] Qu Y.-J. Jia L. Zhang X. Wei H. Yue S.-W. (2016). MAPK pathways are involved in neuropathic pain in rats with chronic compression of the dorsal root ganglion. *Evid.Based Complement. Altern. Med.* 2016 1–8. 10.1155/2016/6153215 27504140 PMC4967678

[B26] Randall L. O. Selitto J. J. (1957). A method for measurement of analgesic activity on inflamed tissue. *Arch. Int. Pharmacodyn. Ther.* 111 409–419. 13471093

[B27] Rivat C. Ballantyne J. (2016). The dark side of opioids in pain management: basic science explains clinical observation. *Pain Rep.* 1:e570. 10.1097/PR9.0000000000000570 29392193 PMC5741356

[B28] Sanna M. D. Ghelardini C. Galeotti N. (2014). Regionally selective activation of ERK and JNK in morphine paradoxical hyperalgesia: a step toward improving opioid pain therapy. *Neuropharmacology* 86 67–77. 10.1016/J.NEUROPHARM.2014.06.007 24950452

[B29] Shimoyama N. Shimoyama M. Inturrisi C. E. Elliott K. J. (1996). Ketamine attenuates and reverses morphine tolerance in rodents. *Anesthesiology* 85 1357–1366. 10.1097/00000542-199612000-00017 8968183

[B30] Stein C. (1993). Peripheral mechanisms of opioid analgesia. *Anesth. Analg.* 76 182–191. 10.1213/00000539-199301000-00031 8380316

[B31] Stein C. Lang L. J. (2009). Peripheral mechanisms of opioid analgesia. *Curr. Opin. Pharmacol.* 9 3–8. 10.1016/j.coph.2008.12.009 19157985

[B32] Van Elstraete A. C. Sitbon P. Trabold F. Mazoit J.-X. Benhamou D. (2005). A single dose of intrathecal morphine in rats induces long-lasting hyperalgesia: the protective effect of prior administration of ketamine. *Anesth. Analg.* 101 1750–1756. 10.1213/01.ANE.0000184136.08194.9B 16301254

[B33] Vivancos G. G. Verri W. A. Cunha T. M. Schivo I. R. S. Parada C. A. Cunha F. Q. (2004). An electronic pressure-meter nociception paw test for rats. *Braz. J. Med. Biol. Res.* 37 391–399. 10.1590/s0100-879x2004000300017 15060709

[B34] Wang Z. Ma W. Chabot J.-G. Quirion R. (2009). Cell-type specific activation of p38 and ERK mediates calcitonin gene-related peptide involvement in tolerance to morphine-induced analgesia. *FASEB J.* 23 2576–2586. 10.1096/fj.08-128348 19299480

[B35] Yalcin I. Charlet A. Freund-Mercier M.-J. Barrot M. Poisbeau P. (2009). Differentiating thermal allodynia and hyperalgesia using dynamic hot and cold plate in rodents. *J. Pain* 10 767–773. 10.1016/j.jpain.2009.01.325 19409860

[B36] Zambelli V. O. Fernandes A. C. D. O. Gutierrez V. P. Ferreira J. C. B. Parada C. A. Mochly-Rosen D. (2014). Peripheral sensitization increases opioid receptor expression and activation by crotalphine in rats. *PLoS One* 9:e90576. 10.1371/journal.pone.0090576 24594607 PMC3942445

[B37] Zimmermann M. (1983). Ethical guidelines for investigations of experimental pain in conscious animals. *Pain* 16 109–110. 10.1016/0304-3959(83)90201-4 6877845

