# Supplementary figures and images for: Mitogen-Activated Protein Kinase Signaling Mediates Morphine Induced-Delayed Hyperalgesia
Source: Front Neurosci. 2019 Sep 20;13:1018. doi: 10.3389/fnins.2019.01018 (PMC6763729; doi:10.3389/fnins.2019.01018)

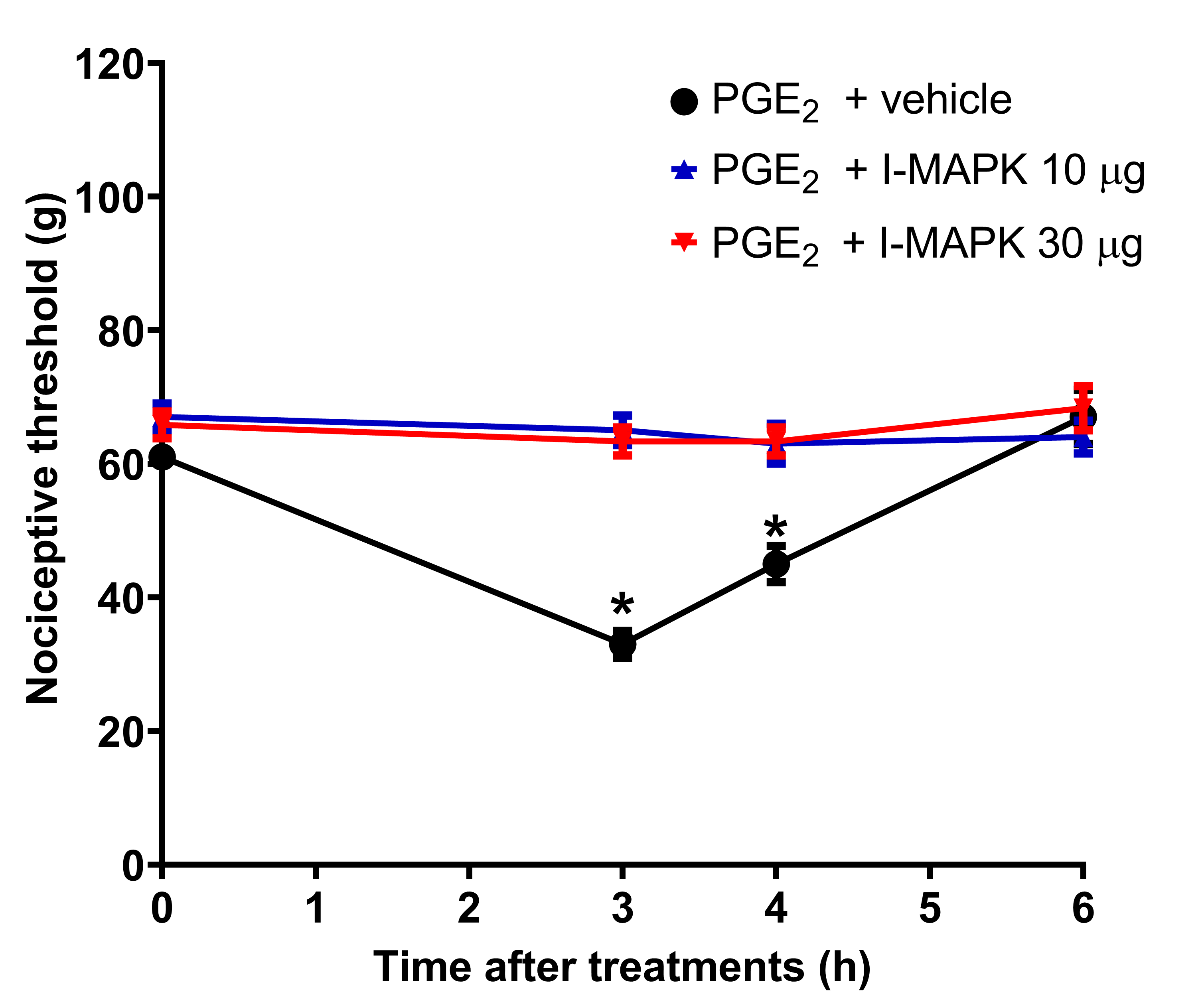

Supplement: FIGURE S1 — Effect of MAPK inhibition on prostaglandin E2-induced hyperalgesia. Nociceptive threshold was obtained in the rat paw pressure test, before (0) and 3, 4, and 6 h after intraplantar PGE2 injection (100 ng/paw). MEK inhibitor (PD 9805) was injected in the paw (30 μg/paw). Data represent mean values ± SEM for six rats per group. ∗ significantly different from baseline (0). Data were analyzed by two-way analysis of variance (ANOVA) with post hoc testing by Tukey. [file Image_1.TIF]

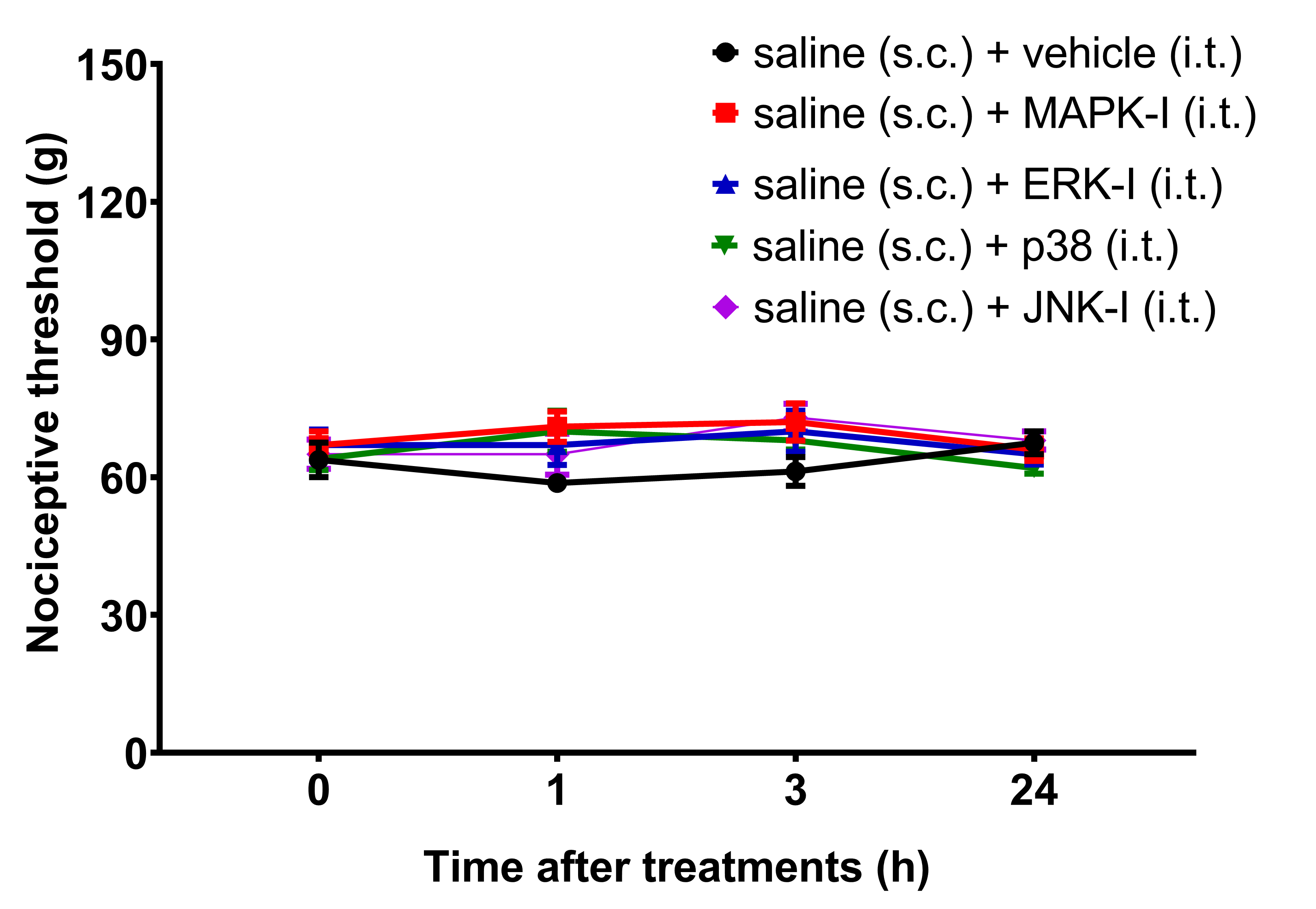

Supplement: FIGURE S2 — Effect of MAPK inhibitors per se on the rat nociceptive threshold. Nociceptive threshold was obtained in the rat paw pressure test, before (0) and 1, 3, and 24 h after subcutaneous intrathecal injection of ERK inhibitor (ERK-I), JNK inhibitor (JNK-I, SP660125), or p38 inhibitor (p38-I, SB20358). Data represent mean values ± SEM for six rats per group. ∗ significantly different from baseline (0). Data were analyzed by two-way analysis of variance (ANOVA) with post hoc testing by Tukey. [file Image_2.TIF]
